# Lower Extremity Skeletal Muscle Mass, but Not Upper Extremity Skeletal Muscle Mass, Is Inversely Associated with Hospitalization in Patients with Type 2 Diabetes

**DOI:** 10.1155/2017/2303467

**Published:** 2017-08-07

**Authors:** Hidetaka Hamasaki

**Affiliations:** ^1^Hamasaki Clinic, Kagoshima, Japan; ^2^Department of Internal Medicine, Kohnodai Hospital, National Center for Global Health and Medicine, Chiba, Japan

## Abstract

**Aim:**

To investigate the association of skeletal muscle mass with metabolic parameters and hospitalization in patients with type 2 diabetes.

**Methods:**

A retrospective observational study was conducted in patients with type 2 diabetes between May 2013 and November 2015. Body composition was measured by bioelectrical impedance analysis. Multiple regression analysis was performed to identify the association between skeletal muscle mass and metabolic parameters. Cox proportional hazard analysis was performed to assess the association between skeletal muscle mass and hospitalization.

**Results:**

A total of 121 patients were enrolled in this study. The mean age of patients was 59.4 ± 14.2 years. During a mean follow-up of 730 ± 253 days, three patients (2.8%) died and 79 patients (65.3%) were admitted to our hospital. After adjustment for age, sex, height, and weight, it was found that lower extremity skeletal muscle mass (LSM) was inversely associated with brachial-ankle pulse wave velocity (*β* = −0.108, *P* = 0.008). Moreover, LSM was significantly associated with reduced risk of hospitalization (hazard ratio = 0.752; 95% confidence interval, 0.601–0.942; *P* = 0.013). In contrast, upper extremity skeletal muscle mass (USM) did not exhibit any significant association.

**Conclusion:**

LSM, but not USM, is important for managing patients with type 2 diabetes. This trial is registered with UMIN000023010.

## 1. Introduction

Decreased strength and impaired function of skeletal muscle are associated with increased mortality [1–3]. Sarcopenia, an age-related decline in muscle mass and function, has a negative impact on quality of life, disability, and mortality in older individuals [4]; thus, prevention of sarcopenia is important. Patients with type 2 diabetes show lower muscle strength [5] than those without type 2 diabetes. A previous study showed that type 2 diabetes is associated with skeletal muscle loss in community-dwelling older individuals [6]. Current evidence suggests that patients with type 2 diabetes have reduced muscle strength and impaired physical function due to hyperglycemia, insulin resistance, ectopic fat in skeletal muscle, and progressed diabetic neuropathy [7]. We have previously reported that lower extremity skeletal muscle mass (LSM) is important for improving metabolic parameters and body composition in obese patients with type 2 diabetes [8, 9]. Moreover, Frank-Wilson et al. [10] showed that lower extremity muscle density is associated with fall risk in older individuals. Guadalupe-Grau et al. [11] have also reported that the maximal isometric strength of knee and hip muscle is a predictor of mortality and hospitalization in women. However, no longitudinal studies have investigated the association between LSM and disease prognosis in patients with type 2 diabetes. The author hypothesizes that LSM plays an important role in managing type 2 diabetes. Hence, the aim of this study was to examine the association of LSM with hospitalization in Japanese patients with type 2 diabetes.

## 2. Methods

### 2.1. Study Design and Patients

A retrospective cohort study was conducted in patients with type 2 diabetes who were treated at the Kohnodai Hospital, National Center for Global Health and Medicine, Japan between May 2013 and November 2015. A total of 121 patients whose body composition was measured at the outpatient clinic were included. Patients were instructed to consume a calorie-restricted diet of 25–30 kcal/kg (ideal body weight) per day, which was prescribed by certified nutritional educators as diet therapy for diabetes, during the study period. All patients were evaluated and followed until death or till the end of follow-up in May 2016. General information, such as date of hospital admission, duration of hospitalization, and cause of hospitalization, was retrieved from the electronic health record of our hospital. The study protocol was approved by the Medical Ethics Committee of the National Center for Global Health and Medicine (reference number NCGM-G-002052), and the study was performed in accordance with the Declaration of Helsinki. The opt-out method of obtaining informed consent was used. The patients were anonymized to protect their personal information.

### 2.2. Anthropometric and Physiological Measurements

Patients' height and weight were measured using a rigid stadiometer (TTM stadiometer; Tsutsumi Co. Ltd., Tokyo, Japan) and calibrated scales (AD-6107NW; A&D Medical Co. Ltd., Tokyo, Japan), respectively. Body mass index (BMI) was calculated as body weight in kilograms divided by the square of body height in meters. Waist circumference was measured at the umbilical level at the end of exhalation in standing position. Blood pressure was measured in sitting position using an automatic sphygmomanometer (HBP-9020; Omron Co. Ltd., Tokyo, Japan). Arterial stiffness was examined by measuring both brachial-ankle pulse wave velocity (baPWV) and heart rate-corrected augmentation index (AIx_75_) using a pulse pressure analyzer (BP-203RPE; Nihon Colin, Tokyo, Japan) and a digital automatic sphygmomanometer (HEM-9000AI; Omron Co. Ltd., Tokyo, Japan), respectively. The average value of right and left baPWV was used for the analysis.

### 2.3. Analyses of Lifestyle and Medical History

Technicians from the Clinical Research Center of the National Center for Global Health and Medicine at Kohnodai Hospital asked patients about their duration of diabetes, smoking status, alcohol consumption, physical activity levels, and history of cardiovascular disease (CVD), such as stroke, nonfatal coronary heart disease, or peripheral artery disease, at the outpatient clinic. To quantify patients' smoking status, the Brinkman index (number of cigarettes per day multiplied by the number of years) was calculated [12]. Patients were also asked about their regular exercise habits using a simple questionnaire. The duration of exercise performed per day was calculated using a physical activity questionnaire, which comprised three items: (1) Do you engage in any physical exercise? If yes, what kind of exercise do you perform regularly?; (2) How often do you exercise in a week?; and (3) How long do you exercise per session?

### 2.4. Blood Examination

Blood samples were taken from the antecubital vein in the morning. Plasma glucose and hemoglobin A1c (HbA1c) levels were measured. Enzymatic methods were used to assess plasma glucose (Glucose Assay Kit; Wako Pure Chemical Industries, Osaka, Japan). HbA1c levels were measured using automated enzyme-linked immunosorbent assays (E-test TOSOH II; Tosoh, Tokyo, Japan).

### 2.5. Analysis of Body Composition

Body composition was analyzed using a bioelectrical impedance analysis device (InBody720; Biospace Co. Ltd, Tokyo, Japan), which uses a patented 8-point tactile electrode system. This method is based on the principle that lean body mass contains higher water and electrolyte content than fatty tissue; hence, these tissues can be distinguished by electrical impedance. The bioelectrical impedance analysis device uses six frequencies (1, 5, 50, 250, 500, and 1000 kHz) to produce 30 impedance values for five body segments, such as right upper extremity, left upper extremity, trunk of the body, right lower extremity, and left lower extremity [13]. A previous study concerning validation of this method has demonstrated that body composition measured using this device is highly correlated with dual-energy X-ray absorptiometry measurements [14]. Body composition analysis was performed within 3 months after acquiring medical history. Upper extremity skeletal muscle mass (USM) and LSM were calculated by summing right and left upper and lower extremity skeletal muscle mass, respectively.

### 2.6. Statistical Analysis

Statistical analyses were performed using SPSS version 24 (IBM Co. Ltd., Chicago, IL, U.S.). All values are expressed as mean ± standard deviation, except for sex. Pearson's correlation coefficient was calculated to analyze the correlation of USM and LSM with clinical data. In addition, multiple regression analysis adjusted for confounding covariates such as age, sex, height, and weight was performed to test the independent association of USM and LSM with clinical parameters. Furthermore, Cox proportional hazards analysis was performed to assess the independent association of USM and LSM with hospitalization. We included age, sex, height, weight, history of CVD, smoking status (Brinkman index), alcohol consumption, exercise time, HbA1c levels, and duration of diabetes in the Cox model. The entry variable was the date of acquiring medical history at the outpatient clinic. Follow-up was at the first day of hospitalization or May 1, 2016, whichever came first. *P* values < 0.05 were considered statistically significant.

## 3. Results

A total of 121 patients (67 men and 54 women) were enrolled in the present study. The mean age of patients was 59.4 ± 14.2 years, and mean BMI was 27.6 ± 6.8 kg/m^2^.  Table 1 shows clinical characteristics of patients. Total skeletal muscle mass, USM, and LSM were inversely correlated with AIx_75_ (*r* = −0.402, *P* = 0.001; *r* = −0.317, *P* = 0.008; and *r* = −0.447, *P* < 0.001, resp.)_._ Similarly, total skeletal muscle mass and LSM were also inversely correlated with baPWV (*r* = −0.239, *P* = 0.022 and *r* = −0.262, *P* = 0.012, resp.). After adjustment for age, sex, height, and weight, LSM was still inversely associated with baPWV (*β* = −0.108, *P* = 0.008); however, such correlations disappeared after further adjustment for age, sex, height, weight, mean blood pressure, and HbA1c levels. No other significant associations between LSM or USM and clinical parameters were observed. During the mean follow-up of 730 ± 253 days, three patients (2.8%) died and 79 patients (65.3%) were admitted to our hospital. Reasons for hospitalization included glycemic control (*n* = 63), gastroenterological disease (*n* = 5), infectious disease (*n* = 5), hepatic disease (*n* = 2), psychoneurological disease (*n* = 2), respiratory disease (*n* = 1), and ocular disease (*n* = 1). Cox proportional hazards analyses adjusted for age, sex, height, weight, history of CVD, Brinkman index, alcohol consumption, exercise time, mean blood pressure, HbA1c levels, and duration of diabetes revealed that LSM had a significant association with hospitalization; however, USM had no such association (Table 2).

## 4. Discussion

The present study demonstrates that LSM is associated with reduced risk of hospitalization. To the best of my knowledge, this is the first study to show that LSM has beneficial impact on hospitalization in patients with type 2 diabetes.

Epidemiological studies have shown that higher muscle mass is associated with reduced risk of cardiovascular and all-cause mortality in elderly population [15] and patients with CVD [16]. Heitmann and Frederiksen [17] also showed that a small thigh circumference is related to an increased risk of developing heart disease and total mortality, suggesting that LSM is particularly important for good health. Although the mechanism underlying the association between LSM and mortality has not been clarified, authors have hypothesized that individuals with decreased LSM are sedentary. Physical inactivity promotes skeletal muscle loss; compared with sedentary individuals, physically active individuals seem to retain their leg strength in old age [18]. Indeed, nonexercise activity thermogenesis besides regular exercise is positively correlated with LSM [8]. Moreover, physical inactivity increases the risk of major noncommunicable diseases, including type 2 diabetes [19]. Locomotive physical activity plays a pivotal role in glycemic control and reduction of mortality risk in patients with type 2 diabetes [20]. The association between LSM and risk of hospitalization suggests that LSM is a prognostic marker of health in patients with type 2 diabetes.

Recently, skeletal muscle has been identified as an endocrine organ, which secrets myokines that communicate with adipose tissue, the liver, the pancreas, the bones, and the brain [21]. Myokines include various bioactive peptides such as IL-6, IL-8, IL-15, fibroblast growth factor-21, brain-derived neurotrophic factor, and irisin; some of them have a potential to improve insulin sensitivity, glucose uptake, and glucose oxidation [22]. In this study, most patients were hospitalized because of hyperglycemia. Skeletal muscle loss may deteriorate glycemic control mediated by myokines. However, to elucidate the effect of skeletal muscle mass on glycemic control, further studies are needed in the future.

In addition, skeletal muscle loss is associated with arterial stiffness, which may result in increased risk of CVD or mortality. Ochi et al. [23] have reported that baPWV is an independent risk factor of sarcopenia after evaluating mid-thigh muscle cross-sectional area in middle-aged to elderly men. Lee et al. [24] have also reported that limb muscle mass measured by bioelectrical impedance analysis is inversely associated with augmentation index in Asian population. Skeletal muscle loss causes insulin resistance and physical inactivity [25]. Similarly, arterial stiffness is closely linked to reduced exercise capacity [26]. Therefore, it is clear that the relationship between skeletal muscle mass, physical activity, and vascular elasticity certainly plays a role in maintaining good health.

Several limitations associated with this study need to be addressed. First, this study could not evaluate the association of skeletal muscle mass with mortality or cardiovascular events. Further investigations with a larger number of patients and longer duration are warranted. Second, although the bioelectrical impedance analysis device used in this study was validated, skeletal muscle mass measured using regression equation might have been influenced by measurement conditions. Third, muscle strength of extremities was not measured in this study. However, a significant positive correlation between muscle mass and strength is well established [27, 28]. Despite these limitations, the present study demonstrates that LSM is important to prevent hospitalization in patients with type 2 diabetes. To ensure this association and assess the effect of LSM on disease prognosis, additional prospective studies are required.

## 5. Conclusion

In conclusion, findings of this study suggest that LSM, but not USM, is essential for good health of patients with type 2 diabetes. Prevention of skeletal muscle loss is important for improving quality of life, disability, and mortality. Leg exercises such as resistance training plus regular walking should be performed to improve LSM in patients with type 2 diabetes. Clinicians should probably pay attention to lower extremity skeletal muscle as well as weight, waist circumference, and BMI for effectively managing type 2 diabetes.

## Figures and Tables

**Table 1:** Characteristics of study subjects.

*Demographics*	
*n*	121
Age (years)	59.4 (14.2)
Sex (men/women)	67/54
Alcohol consumption (g ethanol per day)	21.9 (39.4)
Brinkman index	275.9 (483.6)
Exercise time (min/day)	14.6 (41.4)
History of cardiovascular disease (yes/no)	7/114
Duration of diabetes (years)	7.9 (9.3)
*Anthropometric data*	
Height (cm)	161.7 (10.4)
Weight (kg)	72.4 (20.7)
BMI (kg/m^2^)	27.6 (6.8)
Waist circumference (cm)	95.9 (16.1)
*Body composition*	
Total skeletal muscle mass (kg)	25.3 (6.3)
Upper extremity skeletal muscle mass (kg)	5.0 (1.6)
Lower extremity skeletal muscle mass (kg)	14.4 (3.9)
Body fat mass (kg)	24.3 (12.9)
Body fat percentage (%)	32.6 (10.9)
*Physiological and biochemical data*	
Systolic blood pressure (mmHg)	138.9 (22.9)
Diastolic blood pressure (mmHg)	79.8 (14.3)
Plasma glucose (mg/dL)	178.3 (78.4)
HbA1c (%)	8.5 (2.1)
Brachial-ankle pulse wave velocity (cm/s)	1697 (451)
Augmentation index	74.3 (14.2)

Data are represented as the mean (SD) except for the number of subjects, sex, and history of cardiovascular disease. BMI: body mass index; HbA1c: hemoglobin A1c.

**Table 2:** Cox proportional hazard analysis for evaluating the associations of upper and lower extremity skeletal muscle mass with hospitalization in patients with type 2 diabetes.

	Upper extremity			Lower extremity		
	HR	95% CI	*P*	HR	95% CI	*P*
Age (per 1 year increase)	1.013	0.989–1.037	0.29	1.010	0.987–1.033	0.39
Sex						
Men	0.769	0.322–1.838	0.77	0.749	0.339–1.652	0.47
Women	(ref)			(ref)		
Height (per 1 cm increase)	1.015	0.967–1.065	0.55	1.068	1.001–1.139	0.047
Weight (per 1 kg increase)	1.020	0.990–1.051	0.19	1.030	1.005–1.055	0.017
Smoking (per 1 unit increase in Brinkman index)	1.000	1.000-1.001	0.35	1.000	1.000-1.001	0.39
Alcohol consumption (per 1 g/day increase in ethanol consumption)	1.121	1.001–1.255	0.049	1.110	0.990–1.244	0.073
History of CVD						
Yes	1.119	0.459–2.728	0.8	0.835	0.330–2.117	0.71
No	(ref)			(ref)		
Exercise time (per 1 min/day increase)	0.994	0985–1.004	0.25	0.995	0.986–1.004	0.29
Duration of diabetes (per 1 year increase)	1.015	0.984–1.046	0.35	1.023	0.996–1.050	0.098
Mean blood pressure (per 1 mmHg increase)	0.999	0.986–1.011	0.82	0.998	0.985–1.010	0.71
HbA1c (per 1% increase)	1.348	1.181–1.539	<0.001	1.364	1.193–1.561	<0.001
Skeletal muscle mass (per 1 kg increase)	0.744	0.438–1.263	0.27	0.752	0.601–0.942	0.013

HR: hazard ratio; CI: confidence interval; HbA1c: hemoglobin A1c.
